# Optimizing allocation of curricular content across the Undergraduate & Graduate Medical Education Continuum

**DOI:** 10.1186/s12909-022-03489-2

**Published:** 2022-06-02

**Authors:** Samara B. Ginzburg, Margaret M. Hayes, Brittany L. Ranchoff, Eva Aagaard, Katharyn M. Atkins, Michelle Barnes, Jennifer B. Soep, Andrew C. Yacht, Erik K. Alexander, Richard M. Schwartzstein

**Affiliations:** 1 Donald and Barbara Zucker School of Medicine at Hofstra/Northwell, Hempstead, NY USA; 2 Medical Intensive Care Unit, Beth Israel Deaconess Medical Center, Boston, MA USA; 3 Harvard Medical School in Boston, Boston, MA USA; 4 Beth Israel Deaconess Medical Center, Boston, MA USA; 5 University of Massachusetts, Amherst, MA USA; 6 Washington University School of Medicine, St. Louis, MO USA; 7 Clinical pediatrics and internal medicine University of Illinois College of Medicine, Chicago, IL USA; 8 University of Colorado School of Medicine, Aurora, CO USA; 9 Brigham and Women’s Hospital and Harvard Medical School, Boston, MA USA; 10 Medicine and Medical Education, Harvard Medical School, Boston, MA USA; 11 Division of Pulmonary, Critical Care, and Sleep Medicine, Beth Israel Deaconess Medical Center, Boston, MA USA

**Keywords:** Continuum of education, Social determinants of health, Curriculum

## Abstract

**Background:**

Medical educators struggle to incorporate socio-cultural topics into crowded curricula. The “continuum of learning” includes undergraduate and graduate medical education. Utilizing an exemplar socio-cultural topic, we studied the feasibility of achieving expert consensus among two groups of faculty (experts in medical education and experts in social determinants of health) on which aspects of the topic could be taught during undergraduate versus graduate medical education.

**Methods:**

A modified Delphi method was used to generate expert consensus on which learning objectives of social determinants of health are best taught at each stage of medical education. Delphi respondents included experts in medical education or social determinants of health. A survey was created using nationally published criteria for social determinants of health learning objectives. Respondents were asked 1) which learning objectives were necessary for every physician (irrespective of specialty) to develop competence upon completion of medical training and 2) when the learning objective should be taught. Respondents were also asked an open-ended question on how they made the determination of when in the medical education continuum the learning objective should be taught.

**Results:**

26 out of 55 experts (13 social determinants of health and 13 education experts) responded to all 3 Delphi rounds. Experts evaluated a total of 49 learning objectives and were able to achieve consensus for at least one of the two research questions for 45 of 49 (92%) learning objectives. 50% more learning objectives reached consensus for inclusion in undergraduate (*n* = 21) versus graduate medical education (*n* = 14).

**Conclusions:**

A modified Delphi technique demonstrated that experts could identify key learning objectives of social determinants of health needed by all physicians and allocate content along the undergraduate and graduate medical education continuum. This approach could serve as a model for similar socio-cultural content. Future work should employ a qualitative approach to capture principles utilized by experts when making these decisions.

**Supplementary Information:**

The online version contains supplementary material available at 10.1186/s12909-022-03489-2.

## Background

Changes in care delivery models and a new appreciation for factors that contribute to health outcomes require physician expertise beyond biomedical sciences. Current expectations for physician competence include socio-cultural based expertise [1, 2], such as proficiency in communication skills, population health, interprofessional education and social determinants of health [3], as well as healthcare economics, healthcare quality and safety principles, and healthcare delivery science.

With an increasing number of socio-cultural topics deemed important in medical training, however, medical schools are inundated with requests to address new content without an increase in pre-clerkship curricular or required clerkship time [1]. Faculty experts tend to elevate the importance of their own specific content area [4], fueling a push to include content in excess of what a curriculum can or should handle, which is ultimately detrimental to learning. Frustrated educators may resort to approaches that “check the box” that a topic was addressed in the absence of time necessary to deliver a meaningful learning opportunity.

Although the foundational principles and knowledge for these competencies are recognized as both necessary and important, curriculum developers struggle with selecting which new topics to teach and when to focus on them during the course of medical training. Medical educators frequently reference the continuum of learning across undergraduate (UME) and graduate medical education (GME), yet calls for education in these topics often fall upon an already full UME curriculum [5], even though some content may be more readily understood and applied in the context of the full patient-care responsibilities associated with GME.

The Millennium Conference, one of a series of bi-annual national conferences sponsored by the Carl J. Shapiro Institute for Education and Research at Harvard Medical School and Beth Israel Deaconess Medical Center [6, 7], and co-sponsored by the Association of American Medical Colleges, was convened to address the topic: “*From Student to Doctor: Aligning UME and GME Teaching to Ensure Success*”. Teams of faculty from eight medical schools (Table 1) discussed how educators could address allocation of content across the UME/GME continuum. Conferees recommended that portions of this content would be better aligned with authentic patient responsibility during GME. A post-conference task force was created to develop recommendations to inform educators on best practices for more effective utilization of the UME-GME continuum.

**Table 1:** Millennium Conference “*From Student to Doctor: Aligning UME and GME Teaching to Ensure Success* Medical School Participants

Donald and Barbara Zucker School of Medicine at Hofstra/Northwell	
Harvard Medical School	
Illinois College of Medicine	
NYU Grossman School of Medicine	
Ohio State College of Medicine	
Rush Medical College at Rush University	
The University of Texas Health Science Center at Houston (McGovern Medical School)	
University of Colorado School of Medicine	
University of Nebraska	

This multi-institutional task force, comprising medical education leaders from the eight institutions represented at the conference, applied principles stemming from the conference. The eight schools were selected from 17 that responded to an RFA for the conference. Schools were asked to identify key questions related to the topic, to describe their own work in this area, and to nominate a team of faculty to participate in the conference. Selection of schools was based on the quality of the questions and proposed team as well as the work being done locally to address the topic being discussed; consideration was given to geographic representation and to achieving a mix of private and public schools. Utilizing the Delphi method and an exemplar socio-cultural topic, we studied whether we could (1) reach expert consensus on which aspects of the topic could be taught during UME versus GME, and (2) gain insights into how experts make the decisions about when elements of a socio-cultural topic are taught during the UME/GME continuum.

The task force identified Social Determinants of Health (SDOH) as the exemplar topic to study. SDOH was chosen in part because the LCME requires that it be taught and because national groups have already established Learning Objectives (LOs). Recent content expert consensus on inclusion of SDOH learning objectives in UME recommended that this subject constitute 29% of UME curricula [8] yet it is only one of many socio-cultural topics medical schools are expected to teach [9]. Despite adult learning principles that emphasize the importance of the connection of learning to clinical experiences [10], much of this content is now taught in US medical schools at a time prior to the majority of patient encounters [11].

Faculty members responsible for UME are often siloed from those responsible for GME, which can hinder use of the entirety of an individual’s formal medical education experience. Herein we describe a process, utilizing a national consensus methodology, as an approach to address this problem and report the subsequent results and their implications for determining which portions of socio-cultural topics could be taught in UME and which in GME. We believe this approach may serve as a model for allocating other socio-cultural curricular content areas within medical education.

## Methods

Utilizing the modified Delphi method, a consensus generating process of expert opinion [12], we sought to develop agreement on which LOs for SDOH are best taught at each stage of medical education – UME and GME. We chose the modified Delphi as compared to the Delphi to accommodate the geographic dispersion of the experts participating in the process.

### Survey creation

The LOs for SDOH were compiled from two published expert education resources on the topic, specifically the Association of American Medical Colleges’ Tool for Assessing Culture Competence Training (TACCT), a 42-item list of LOs divided into 6 domains [13], and the Greater New York Health Association’s 25 item list of LOs divided into 5 domains [14]. These LOs were combined, duplicates removed, and slight changes to the wording (e.g., to change phrasing of objectives to incorporate active learning verbs where necessary) were made. A survey was created with a total of 39 objectives grouped into 5 domains (see Additional file 1). These objectives as well as other survey demographic questions were created and revised in an iterative fashion by task force members. Cognitive interviewing and pilot testing were done according to best practices of survey design [15] and the survey was revised accordingly. Cognitive interviewing is an evidence-based tool that can help survey developers collect validity evidence based on survey content and the thought processes participants engage in while answering survey questions.

The survey respondents included two panels: 1) experts in medical education, and 2) experts in SDOH. Subject matter experts are needed to identify content, but they tend to be passionate about their topic [15], which may lead to an unconscious bias toward assigning all content a high priority status. Medical education experts were included to provide perspective on when the content would be most developmentally relevant and to prioritize the topics for learners, in the context of proximity to patient care responsibilities and the reality of a zero-sum curriculum, which often necessitates removing other content when new material is inserted. We hypothesized that the two expert groups would differ on their characterization of LOs as “essential” and on the timing of the allocation of topics within the curricula.

We identified SDOH experts from across the country. Authors (BLR, MMH) performed an online search on each suggested expert to ensure they had demonstrated expertise in the field. All content experts were discussed and reviewed by authors (SBG, EKA, MMH, RMS). The medical education experts consisted of Millennium Conference attendees, all chosen initially by their schools to participate in the Conference based on their expertise as educators, who were not part of this task force.

Surveys were disseminated online through the Qualtrics (Provo, UT) platform via an anonymous email survey link with built-in survey response reminders for each round. A series of surveys were administered over two-week periods from January 2019 to March 2019. In each survey, respondents were presented with the list of LOs and asked to determine if the LO was one that every physician (irrespective of specialty/career path) must be able to know/do at the completion of medical training. Respondents used a 2-point rating system: (1) Yes (this is essential) (2) No (this is not essential). If “yes” was selected, respondents were asked if the LO should primarily be taught during UME or GME. Additionally, survey respondents were given the opportunity to suggest alternative wording for LOs and propose additional objectives. Respondents were also asked an open-ended question on how they made the determination of where in the medical education continuum (UME vs. GME) the LO should be taught.

Our taskforce decided a priori that there would be a minimum of two and a maximum of four online survey rounds. A threshold of ≥70% percent agreement was used to define consensus, in accordance with accepted Delphi practice [16], for identifying a LO as essential, as well as determining when in the continuum of medical education (UME vs. GME) it should be taught. LOs that achieved consensus for both parameters were removed and not included in subsequent rounds. For rounds 2 and 3, LOs for which consensus had not been achieved, as well as newly suggested LOs, modified LOs, and those that did not reach consensus for being essential were included. Task force participants reviewed the data after each round to determine if suggested word changes and/or new LOs, would be added to the next round. After the initial round, LO results and summary statistics were distributed to respondents before they were asked to complete the next survey.

Descriptive statistics were conducted for demographics and the percent agreement between LOs. We analyzed all Delphi survey data using JMP© 14 (SAS Institute, Cary, NC). This study was determined to be exempt by the institutional review board at Beth Israel Deaconess Medical Center (Boston, MA).

## Results

Fifty-five experts (21 SDOH experts and 34 education experts) were contacted. Of those, 38 (16 SDOH experts and 22 education experts) agreed to participate in the Delphi process (Table 2). For the first round, 33 of 38 experts participated (response rate 87%). Of these, 16 were SDOH experts and 17 were education experts. SDOH experts’ areas of focus included economic stability, education, health and healthcare, neighborhood and built environment, and social and community context. Education experts’ areas of focus included continuing medical education, medical student education, graduate medical education, and multicultural affairs/equity/diversity and inclusion.

**Table 2:** Panelist Demographic Data, *n* = 33

Variables	% (n)
**Gender**	
Female	48.5 (16)
**Race/Ethnicity**	
Asian	12.12 (4)
Black or African American	12.12 (4)
Hispanic	15.15 (5)
White or Caucasian	63.64 (21)
**Degree**	
MD	78.8 (26)
PhD	18.18 (6)
EdD	3.03 (1)
Masters	27.27 (9)
JD	3.0 (1)
**Institutions Represented**	17
**Experts**	
Education Expert	51.5 (17)
Social Determinants of Health Experts	48.5 (16)
**Primary professional activity of experts, n = 33**	
Administrator	27.3 (9)
Clinical care	21.2 (7)
Community activist/engagement	6.1 (2)
Policy advocate	3.0 (1)
Researcher	12.1 (4)
Teacher/educator	30.3 (10)

During round 2, 30 of 33 experts (91%) completed the survey: 15 of these were SDOH experts and 15 were education experts. For round 3, 26 out of 30 experts responded. Of these, 13 were SDOH experts and 13 were education experts.

In addition to the original 39 LOs, seven more were added during round 2, and three more were added during round 3, comprising a total of 49 LOs that survey respondents evaluated. Experts were able to achieve consensus for at least one of the two questions they were tasked to answer for 45 of the 49 (92%) LOs (see Table 3). There were 35 (71%) LOs that achieved consensus for inclusion both as an essential LO as well as for inclusion in UME vs. GME: 21 reached consensus with respect to whether it should be taught in UME and 14 in GME. There were seven (14%) LOs that achieved consensus for inclusion as an essential LO; however, experts could not achieve consensus on when these seven LOs should be taught. There were four LOs (8%) that did not reach consensus as an essential LO, i.e., they were deemed not to be essential for all doctors.

**Table 3:** Social Determinants of Health Learning Objectives Level of Consensus

Learning Objective, ***N*** = 49		Consensus reached to be learning objective	Consensus reached on where to be taught
1	Define race, ethnicity and culture, and how they relate to health	Yes	UME
**2**	Describe examples of social determinants of health	Yes	UME
**3**	Describe the challenges in serving diverse communities	Yes	UME
**4**	Differentiate “equity” from “equality”	Yes	UME
**5**	Understand how common social needs can impact the health of an individual	Yes	UME
**6**	Characterize key areas of disparities at the level of an individual patient	Yes	Not achieved
**7**	Identify patterns of national data demonstrating health disparities	Yes	UME
**8**	Use national resources to support policies that are intended to improve health disparities (such as Healthy People 2020)	Not achieved	Not achieved
**9**	Develop the skills to critically appraise the literature on health disparities	Yes	UME
**10**	Utilize the available research on health disparities to change one’s practice	Yes	GME
**11**	Describe how social determinants of health fit into broader health care policy	Yes	UME
**12**	Recognize disparities of health that are amendable to intervention	Yes	UME
**13**	Develop strategies to promote the elimination of disparities	Yes	Not achieved
**14**	Among colleagues or other individuals, discuss barriers to eliminate health disparities	Yes	GME
**15**	Identify examples of cultural differences within one’s practice’s patient population	Yes	GME
**16**	Recognize patient’s health traditions and beliefs within one’s practice’s patient population	Yes	GME
**17**	Identify community leaders and key stakeholders	No	–
**18**	Collaborate with community leaders to propose a community-based health intervention	No	–
**19**	Utilize cross-cultural communication models	Yes	UME
**20**	Describe the medical neighborhood and the role of community-based organizations within it	Not achieved	Not achieved
**21**	Identify common social needs within the community served by one’s practice	Yes	GME
**22**	Recognize the prevalence of chronic diseases within the community served	Yes	GME
**23**	Identify several local community-based organizations that address specific social needs for patients	Yes	GME
**24**	Identify referral mechanisms for community-based organizations	Yes	GME
**25**	Demonstrate strategies to address/reduce bias in oneself	Yes	UME
**26**	Demonstrate strategies to reduce bias in others	Yes	UME
**27**	Utilize screening tools in your clinical setting to assess patients for social needs that impact health	Yes	GME
**28**	Understand the impact of social determinants of health on patients’ adherence to medical recommendations in one’s clinical setting	Yes	GME
**29**	In the clinical environment, use non-judgmental listening to health beliefs	Yes	UME
**30**	Demonstrate the ability to utilize an interpreter to maximize communication in one’s clinical setting	Yes	Not achieved
**31**	Identify examples of cultural differences within one’s patient population	Yes	Not achieved
**32**	Demonstrate respect and address cultural differences within one’s patient population	Yes	Not achieved
**33**	Identify resources within the health care system, clinical practice, school departments and infrastructure which can help address social determinants of health	Yes	GME
**34**	Recognize the impact of health policy on medicine and health outcomes	Yes	UME
**35**	Describe key features of the legislative process through which physicians can encourage equity and promote health	Not achieved	Not achieved
**36**	Utilize clinical resources to advocate for individual patients and families within clinical encounters	Yes	GME
**37**	Develop the skills to communicate with legislators via e-mail, letter writing, and in-person advocacy	No	–
**38**	Develop the skills to reflect upon one’s own beliefs	Yes	UME
**39**	Identify the value of addressing personal bias	Yes	UME
**40**	Define race and describe how racism and historical discrimination contribute to health disparities	Yes	UME
**41**	Define “privilege” as it is used in discussions of social and racial inequities and how it contributes to health disparities	Yes	UME
**42**	Describe the impact of health literacy on patient health and illness	Yes	UME
**43**	Create strategies to address health literacy in one’s clinical practice	Yes	GME
**44**	Define and identify microaggressions	Yes	UME
**45**	Develop strategies to prevent and address microaggressions in the clinical workplace	Yes	Not achieved
**46**	Define the concept of “identity” and the factors that contribute to forming one’s identity	Not achieved	Not achieved
**47**	Describe the importance of addressing prejudice as part of one’s professional responsibility	Yes	UME
**48**	Discuss social determinants of health with patients to facilitate care	Yes	Not achieved
**49**	Describe potential solutions to address healthcare disparities within one’s community	Yes	GME

Table 4 includes a sample of responses to the open-ended question asking how experts made determinations about where in the continuum a LO should be included.

**Table 4:** Allocating learning objectives into UME vs. GME

Round 1	Round 2
For the majority of these objectives, I don’t think its UME OR GME. Most should be introduced in UME and reinforced/further refined/further developed in a specialty specific way in GME.	Each can be addressed in UME - but will need re-addressing and contextualization in GME.
I had a hard time saying that any were non-essential, and a hard time ‘pushing’ things to GME. I wanted to keep most in UME to at least some extent!	Much of the content should begin to be delivered during UME when professional identity formation and early clinical skills/practice styles are beginning to be developed. However, the SKILLS that we begin teaching should continue and be refined. My concern is that if we begin exposure and clinical skills development too late, that they will already develop “bad habits” that will be hard to re-shape at the GME level.
Many of these should be introduced in UME and reinforced in GME after the trainee has some experience as a provider. For example, definitions should be introduced in UME but revisited with deeper discussion during GME.	
It is difficult to choose when to primarily to teach all skills - in the ideal world all would be introduced in undergraduate medical education and readdressed at the graduate level.	

## Discussion

Utilizing SDOH as a model, our study demonstrated that a modified Delphi technique could harness experts’ abilities to consider UME and GME as an educational continuum, and thereby define which objectives are best taught at which time points.

This is the first time to our knowledge that a study has described, tested and quantified the challenge of assigning content on a particular non-biomedical topic into either UME or GME, a challenge that educators face in practice daily. Although SDOH was used as the exemplar, this exercise has broad applicability for use by medical educators charged with analyzing other content areas for inclusion in UME or GME curricula.

This study was stimulated by the growing list of socio-cultural topics to be covered during training. However, in reflecting on our outcomes and lessons learned, we believe this process may be applicable to any topic, whether biomedical or socio-cultural in which there is controversy about the appropriate time for the teaching to occur.

Our combined group of medical education and SDOH content experts were able to achieve consensus on the usage of 92% (45/49) of the LOs within the continuum of medical education. This confirms that it is not difficult for experts to look at a topic or LO and decide that it is something that should be taught. The group had more difficulty, however, determining where and when in the continuum each LO should be taught, but nevertheless reached consensus for 83% (35/42) of LOs, providing evidence of the value of forced choice methodology through a modified Delphi process [17]. Forced-choice tasks are thought to improve early attentional processes which then allow improved perceptual processing of stimuli [18].

The fact that participants had some difficulty making decisions and were not able to articulate what exact criteria (see Table 4) they used to make these decisions does not lessen the value of the consensus achieved. The group could not achieve consensus on where in the continuum of medical education to place seven (17%) LOs. This may reflect differences between content and educational experts but likely also reflects differences in priorities and opportunities within specific medical school and residency curricula. In general, content experts wanted these LOs to be taught earlier (during UME) whereas education experts wanted these LOs taught during GME. This is consistent with our cross-institutional experiences that content experts, across disciplines, seek to frontload education topics into UME, perhaps as a means of emphasizing their importance, rather than utilizing the full continuum. In addition, UME pedagogy is historically more classroom-based than GME learning. Nevertheless, GME has accommodated formal training of topics like quality and safety into resident conference schedules and rotations. Formal didactics are not as frequent in GME as in UME, but they do exist. To the extent that these topics are considered essential by accreditation bodies, programs must address them for all learners despite the fact that residents and fellows often branch out to individual interests in their clinical pursuits. Pairing basic scientists and clinicians to make discriminating decisions about including content in a curriculum has proven successful [19] and might be a useful paradigm to apply to content and education experts.

Those topics that contained a reference to clinical practice were all allocated to GME. While intuitive on the surface, given the full-time clinical environment of GME, it raises questions as to why none was selected for UME given that most UME curricula now involve direct patient care beginning in the first year of school. It may be that a majority of educators still associate clinical practice most closely with GME, that the core clinical year is so focused on acquiring basic clinical skills it cannot assume responsibility for additional content, or that some of the objectives are indeed best accomplished when the learner is responsible for the patient’s care in the hospital and has achieved some degree of competence in clinical practice.

Items that experts agreed should not be included as part of either UME or GME curricula related to working with stakeholders external to medicine (community leaders, legislators). It is possible that some of these topics may be considered essential for particular specialties or may be part of a niche for individuals with a specific career focus.

There were 50% more LOs (*n* = 21 vs. 14) that were allocated to UME as compared to GME. This may reflect the relative distribution of structured education during UME versus GME. Nevertheless, these results suggest a feasible mapping can be achieved that balances LOs to be covered during UME vs. GME, within the context of an educational continuum. We believe the following factors were crucial to the success of this project: 1) include **both** content and medical education experts, 2) when considering content, pose the question **in this format**: “Is the content essential/not essential for **all** doctors, irrespective of specialty?”, 3) **frame the task** with the lens of the entire UME/GME continuum, and 4) **compel** the participants to allocate topic areas into either the UME **or** GME educational sphere.

Limitations of our modified Delphi study include the following considerations. In the final cohort of those surveyed, there were more experts in medical education than SDOH. The education experts in this study represent a subset of highly invested educators who were selected to participate in the Millennium Conference, and the task of allocating LOs might be more challenging for a general group of medical educators. In addition, we focused on placing a particular LO in only one phase of training, but recognize the value of a spiraled curriculum in which key concepts are revisited one or more times during an educational experience [20]. The task force selected SDOH as the exemplar socio-cultural content area, and this process may not be generalizable to other socio-cultural content areas. Finally, our Delphi study is limited in that it did not offer opportunities for in person discussion with experts to explore and better understand their underlying thought processes, a format which could be used within a single institution.

## Conclusions

In summary, the results of this modified Delphi study demonstrate that it is possible for groups of experts in a particular content area and general medical education to consider UME/GME as an educational continuum and make decisions about the identification of specific content to include and the allocation of that content across the continuum, a process that offers two major benefits: 1) learning objectives are better aligned with maturity of the learner and clinical responsibility, and 2) crowding of the UME curriculum is reduced. Future work should focus on capturing principles utilized by experts to make decisions about what content to include/exclude and where in the continuum to address it. A qualitative approach to capturing this information, e.g., use of focus groups, would be helpful. Utilizing the full UME/GME continuum to allocate content, be it socio-cultural or biomedical, is a difficult but necessary task with which many educators struggle as the demand on time/space in the UME curriculum grows. Collectively, the education community should strive to develop strategies to guide this important work. Finally, although the research focus of this study was to test a process for utilizing the full UME-GME continuum, the results in Table 3 can serve as a timely resource for medical educators seeking to adjust UME-GME curricula to include SDOH and antiracism training in the wake of the COVID-19 pandemic and a national call for social justice.

## Supplementary Information

**Additional file 1.**

## Data Availability

Data gathered from survey was collecting through Qulatrics. No identifying information was recorded. Survey results were kept in password protected Dropbox on password protected computers. The datasets used and/or analyzed during the current study available from the corresponding author on reasonable request.

## Abbreviations

COVID-19: Coronavirus disease 2019
GME: Graduate medical education
LO: Learning objective
SDOH: Social determinants of health
TACCT: Tool for assessing culture competence training
UME: Undergraduate medical education
