# Supplementary material for: Optimizing allocation of curricular content across the Undergraduate & Graduate Medical Education Continuum
Source: BMC Med Educ. 2022 Jun 2;22:425. doi: 10.1186/s12909-022-03489-2 (PMC9161628; doi:10.1186/s12909-022-03489-2)
Supplement: Supplementary file 1 — Additional file 1. [file 12909_2022_3489_MOESM1_ESM.docx]

Appendix 1. Optimizing Allocation of Curricular Content Across the Undergraduate and Graduate Medical Education Continuum-Survey

**Welcome to Round #1 of Social Determinants of Health Delphi Study!**

Thank you for your participation!

The purpose of this study is to develop a consensus on the essential elements of social determinants of health (SDOH) and when they should be taught to medical trainees (i.e., in an undergraduate medical curriculum or graduate medical curriculum).

This survey, including instructions, should take **no longer than 15 minutes to complete**. Please read the following information carefully.
 
This study has been deemed exempt by the Beth Israel Deaconess Medical Center (BIDMC) IRB.

**Instructions:**

You will be presented with a list of social determinants of health learning objectives that have been compiled from the [Greater New York Hospital Association Training Primary Care Residents on Social Determinants of Health](https://hms.az1.qualtrics.com/CP/File.php?F=F_82H5SoA6ruTyNrn%22%20target=%22_blank) and the [AAMC Tool for Assessing Cultural Competence Training (TACCT)](https://hms.az1.qualtrics.com/CP/File.php?F=F_bC9MqIE73qzBvO5%22%20target=%22_blank). For each learning objective, you will be asked to indicate whether this is something that **EVERY** physician (irrespective of specialty/career path) **MUST** know/be able to do at the completion of training to become an independent practitioner regardless of specialty using a 2-point rating system: 
**1-YES (this is essential)**
**2-NO (this is not essential)**
 
If you choose **YES**, you will be asked if this learning objective should be **PRIMARILY** taught during Undergraduate Medical Education (UME) or Graduate Medical Education (GME). You will also be given the opportunity to suggest both alternative wording and additional learning objectives to be included. 
 
As a reminder, your participation is much appreciated but completely voluntary. Your responses will be kept confidential and are anonymous; your name and email address are not linked in any way to your answers. After completing this survey, you will be directed to an independent survey link and asked to provide your name. This information will be used for the purpose of keeping track of participants and will not be linked to the answers you provide in this survey. 
 
Your completion of this survey will serve as your consent to participate in this research study.

If you have questions about the study at any point, please contact study PI, Margaret M. Hayes, MD at mhayes7@bidmc.harvard.edu. Thank you for your time and participation. 
Please note: You will be unable to go back to the previous page once you go to the next page.

**1.** Please indicate the **first letter of your birth month** and the **last four digits of your cell phone number** in order to anonymously match your answers in each round of the Delphi study. (i.e., if you were born in June and your cell phone is 617-122-1203 you would write J1203).

First letter of your birth month ________________________________________________

Last four digits of your cell phone number _______________________________________

**2.** Below is a list of learning objectives. For each one, please indicate if it is something that **EVERY** physician (irrespective of specialty/career path) **MUST** know/be able to do at the completion of training to become an independent practitioner. Please select either **YES** or **NO**.

|  | **YES** | **NO** |
| --- | --- | --- |
| Define race, ethnicity and culture, and how they relate to health | ⭘ | ⭘ |
| Describe examples of social determinants of health | ⭘ | ⭘ |
| Describe the challenges of serving diverse communities | ⭘ | ⭘ |
| Differentiate "equity" from "equality" | ⭘ | ⭘ |
| Understand how common social needs can impact the health of an individual | ⭘ | ⭘ |
| Characterize key areas of disparities at the level of an individual patient | ⭘ | ⭘ |
| Identify patterns of national data demonstrating health disparities | ⭘ | ⭘ |
| Learn how to use national resources to improve health disparities (such as Healthy People 2020) | ⭘ | ⭘ |
| Develop the skills to critically appraise the literature on health disparities | ⭘ | ⭘ |
| Utilize the available research on health disparities to change one's practice | ⭘ | ⭘ |
| Describe how social determinants of health fit into broader health care policy | ⭘ | ⭘ |
| Learn how to recognize disparities of health that are amenable to intervention | ⭘ | ⭘ |
| Develop strategies to promote the elimination of disparities | ⭘ | ⭘ |
| Among colleagues or other individuals, discuss barriers to eliminate health disparities | ⭘ | ⭘ |
| Identify examples of cultural differences within one's practice's patient population | ⭘ | ⭘ |
| Recognize patient's health traditions and beliefs within one's practice's patient population | ⭘ | ⭘ |
| Identify community leaders and key stakeholders | ⭘ | ⭘ |
| Collaborate with community leaders to propose a community-based health intervention | ⭘ | ⭘ |
| Utilize cross-cultural communication models | ⭘ | ⭘ |
| Describe the medical neighborhood and the role of community-based organizations within it | ⭘ | ⭘ |
| Identify common social needs within the community served by one's practice | ⭘ | ⭘ |
| Recognize the prevalence of chronic diseases within the community served | ⭘ | ⭘ |
| Identify several local community-based organizations that address specific social needs for patients | ⭘ | ⭘ |
| Identify referral mechanisms for community-based organizations | ⭘ | ⭘ |
| Demonstrate strategies to address/reduce bias in oneself | ⭘ | ⭘ |
| Demonstrate strategies to reduce bias in others | ⭘ | ⭘ |
| Utilize screening tools in your clinical setting to assess patients for social needs that impact health | ⭘ | ⭘ |
| Understand the impact of social determinants of health on patients adherence to medical recommendations in one's clinical setting | ⭘ | ⭘ |
| In the clinical environment, use non-judgmental listening to health beliefs | ⭘ | ⭘ |
| Demonstrate the ability to utilize an interpreter to maximize communication in one's clinical setting | ⭘ | ⭘ |
| Identify examples of cultural differences within one's patient population | ⭘ | ⭘ |
| Demonstrate respect and address cultural differences within one's patient population | ⭘ | ⭘ |
| Identify health care system, clinical practice or school departments and infrastructure which can help address social determinants of health | ⭘ | ⭘ |
| Recognize the impact of health policy on medicine and health outcomes | ⭘ | ⭘ |

| Describe key features of the legislative process through which physicians can encourage equity and promote health | ⭘ | ⭘ |
| --- | --- | --- |
| Appreciate the power of advocating for individual patients and families within clinical encounters via engagement with various clinical resources | ⭘ | ⭘ |
| Develop the skills to communicate with legislators via e-mail, letter writing, and in-person advocacy | ⭘ | ⭘ |
| Develop the skills to reflect upon one's own beliefs | ⭘ | ⭘ |
| Identify the value of addressing personal bias | ⭘ | ⭘ |

**3.** Below is the list of learning objectives you indicated that **EVERY** physician (irrespective of specialty/career path) **MUST** know (from the last question). Please indicate for each learning objective if it should be **PRIMARILY** taught during Undergraduate Medical Education (UME) or Graduate Medical Education (GME). Please select one or the other.

|  | **UME** | **GME** |
| --- | --- | --- |
| Define race, ethnicity and culture, and how they relate to health | ⭘ | ⭘ |
| Describe examples of social determinants of health | ⭘ | ⭘ |
| Describe the challenges of serving diverse communities | ⭘ | ⭘ |
| Differentiate "equity" from "equality" | ⭘ | ⭘ |
| Understand how common social needs can impact the health of an individual | ⭘ | ⭘ |
| Characterize key areas of disparities at the level of an individual patient | ⭘ | ⭘ |
| Identify patterns of national data demonstrating health disparities | ⭘ | ⭘ |
| Learn how to use national resources to improve health disparities (such as Healthy People 2020) | ⭘ | ⭘ |
| Develop the skills to critically appraise the literature on health disparities | ⭘ | ⭘ |
| Utilize the available research on health disparities to change one's practice | ⭘ | ⭘ |
| Describe how social determinants of health fit into broader health care policy | ⭘ | ⭘ |
| Learn how to recognize disparities of health that are amenable to intervention | ⭘ | ⭘ |
| Develop strategies to promote the elimination of disparities | ⭘ | ⭘ |
| Among colleagues or other individuals, discuss barriers to eliminate health disparities | ⭘ | ⭘ |
| Identify examples of cultural differences within one's practice's patient population | ⭘ | ⭘ |
| Recognize patient's health traditions and beliefs within one's practice's patient population | ⭘ | ⭘ |
| Identify community leaders and key stakeholders | ⭘ | ⭘ |
| Collaborate with community leaders to propose a community-based health intervention | ⭘ | ⭘ |
| Utilize cross-cultural communication models | ⭘ | ⭘ |
| Describe the medical neighborhood and the role of community-based organizations within it | ⭘ | ⭘ |
| Identify common social needs within the community served by one's practice | ⭘ | ⭘ |
| Recognize the prevalence of chronic diseases within the community served | ⭘ | ⭘ |
| Identify several local community-based organizations that address specific social needs for patients | ⭘ | ⭘ |
| Identify referral mechanisms for community-based organizations | ⭘ | ⭘ |
| Demonstrate strategies to address/reduce bias in oneself | ⭘ | ⭘ |

| Demonstrate strategies to reduce bias in others | ⭘ | ⭘ |
| --- | --- | --- |
| Utilize screening tools in your clinical setting to assess patients for social needs that impact health | ⭘ | ⭘ |
| Understand the impact of social determinants of health on patients adherence to medical recommendations in one's clinical setting | ⭘ | ⭘ |
| In the clinical environment, use non-judgmental listening to health beliefs | ⭘ | ⭘ |
| Demonstrate the ability to utilize an interpreter to maximize communication in one's clinical setting | ⭘ | ⭘ |
| Identify examples of cultural differences within one's patient population | ⭘ | ⭘ |
| Demonstrate respect and address cultural differences within one's patient population | ⭘ | ⭘ |
| Identify health care system, clinical practice or school departments and infrastructure which can help address social determinants of health | ⭘ | ⭘ |
| Recognize the impact of health policy on medicine and health outcomes | ⭘ | ⭘ |
| Describe key features of the legislative process through which physicians can encourage equity and promote health | ⭘ | ⭘ |
| Appreciate the power of advocating for individual patients and families within clinical encounters via engagement with various clinical resources | ⭘ | ⭘ |
| Develop the skills to communicate with legislators via e-mail, letter writing, and in-person advocacy | ⭘ | ⭘ |
| Develop the skills to reflect upon one's own beliefs | ⭘ | ⭘ |
| Identify the value of addressing personal bias | ⭘ | ⭘ |

**4.** Please suggest any changes to the wording in the above learning objectives. To see full list, please click on this link: [Learning Objectives of Social Determinants of Health](https://hms.az1.qualtrics.com/CP/File.php?F=F_1G1tU1wfTmwyuwt%22%20target=%22_blank).

_____________________________________________________________________________________

_____________________________________________________________________________________

**5.** Please suggest any additional learning objectives for social determinants of health to be rated in the next round.

_____________________________________________________________________________________

_____________________________________________________________________________________

**6.** If you have any additional thoughts, comments, or suggestions, please include them here.

_____________________________________________________________________________________

_____________________________________________________________________________________

**7.** Please comment on what principles/criteria you utilized to determine if an objective should be taught during UME versus GME.

_____________________________________________________________________________________

_____________________________________________________________________________________

**8.** What is your gender?

- Male
- Female
- Non-binary/third gender
- Prefer to self-describe ____________________________________________________________
- Prefer not to answer

**9.** What is your race/ethnicity? **Check all that apply.**

- American Indian or Alaska Native
- Asian
- Black or African American
- Hispanic
- Native Hawaiian or Pacific Islander
- White/Caucasian
- Other(s) (write in) _________________

**10.** What is your degree(s)? **Check all that apply.**

- MD
- DO
- PhD
- Pharm.D
- Psy.D
- RN
- NP or PA
- MPH
- MPA
- EdD
- MEd or equivalent
- MBA
- Other(s) (write in) _________________

**11.** What institution are you primarily affiliated with?

- Association of American Medical College
- Donald and Barbara Zucker School of Medicine at Hofstra/Northwell
- George Washington University Milken Institute of Public Health
- Harvard Medical School
- John P. and Katherine G. McGovern Medical School
- New York University School of Medicine
- Northwestern University Feinberg School of Medicine
- Ohio State University College of Medicine
- Rush Medical College
- Stanford University School of Medicine
- University of California San Francisco School of Medicine
- University of Cincinnati College of Medicine
- University of Colorado School of Medicine
- University of Illinois College of Medicine
- University of Missouri Kansas City School of Medicine
- University of Nebraska College of Medicine
- University of Pennsylvania Perelman School of Medicine
- University of Toronto's Faculty of Medicine
- Washington University School of Medicine
- Other (write in) _________________________________________________________________

**12.** How do you primarily define yourself?

- Education Expert
- Social Determinants of Health Expert
- Both an Education and Social Determinants of Health Expert

**13.** What are your leadership role(s) within your institution? **Check all that apply.**

- Continuing medical education (e.g., CME course director)
- Dean or associate dean
- Medical student education (e.g., course director, clerkship director)
- Resident core teaching faculty or elective/rotation/course director
- Resident education or fellow education (e.g., program director, site director, associate or assistant director)
- Other(s) (write-in) _______________________________________________________________

**14.** What are your academic role(s) within your institution? **Check all that apply.**

- Continuing medical education (e.g., dean or assistant/associate dean for CME/CPD, CME director or course director)
- Graduate medical education (e.g., DIO, GME program director, dean or assistant/associate dean, vice chair of education in your clinical department)
- Medical student affairs (e.g., dean or assistant/associate dean for student affairs, or student affairs director)
- Medical student education (e.g., dean or assistant/associate dean for curriculum/medical education, course director, clerkship director)
- Multicultural affairs/diversity/inclusion officer (e.g., dean or assistant/associate dean for multicultural affairs/diversity of inclusion, or director)
- Public health officer (e.g., director or assistant/associate director, commissioner)
- Public health or health service research center director (e.g., assistant/associate director, vice chair of research)
- Research education (e.g., MPH or equivalent program director or assistant/associate director, dean or assistant/associate dean)
- Resident or fellow education (e.g., program director, site director, rotation director, associate or assistant director)
- Other(s) (write in) _______________________________________________________________

**15.** What is your area of expertise within social determinants of health? **Check all that apply.**

- Economic Stability (e.g., employment; food insecurity; housing instability; poverty)
- Education (e.g., early childhood education and development; enrollment in higher education; high school graduation; language and literacy)
- Health and Health Care (e.g., access to health care; access to primary care; health literacy)
- Neighborhood and Built Environment (e.g., access to foods that support healthy eating patterns; crime and violence; environmental conditions; quality of housing)
- Social and Community Context (e.g., civic participation; discrimination; incarceration; social cohesion)
- Other(s) (write in) _______________________________________________________________

**16.** Which best describes your primary (>50% of your time) professional activity?

- Administrator
- Clinical care
- Community activist/engagement
- Policy advocate
- Researcher
- Teacher/educator
- Other (write in) ________________________________________________________________

You have reached the end of the survey! When you click the next arrow, you will be redirected to another survey that will ask you to provide your name. This information will allow us to track response rates for each round of the Delphi process. **There are no links between the two surveys.** Thanks again for your input!
